# Research on the Antiaging Activity of Licorice Water Extract in Aging Mice via Antioxidation, Neuronal Protection, Gut Microbiota Restoration, and PI3K/AKT/mTOR Modulation

**DOI:** 10.1155/mi/8844859

**Published:** 2025-11-19

**Authors:** Yanhua Yu, Baiji Xue, Tong Liu, Xianwen Yue, Dawei Liu, Xia Yu, Yang Xu, Xueliang Zhao, Xuefeng Li

**Affiliations:** ^1^College of Pharmacy, Baicheng Medical College, Baicheng 137000, Jilin, China; ^2^Cancer Center, The First Hospital of Jilin University, Changchun 130021, Jilin, China

**Keywords:** antiaging, antioxidant activities, gut microbiota, licorice, neuronal protection, PI3K-AKT pathway

## Abstract

**Background:**

Aging is a multifaceted physiological process characterized by progressive multiorgan dysfunction, oxidative stress, neuronal injury, cognitive impairment, and alterations in gut microbiota composition. Licorice, a widely used traditional medicinal herb, contains diverse bioactive constituents; however, its overall antiaging properties and mechanistic basis in aging models have not been systematically elucidated.

**Methods:**

Aging mice model was established using D-galactose (D-Gal). Body weight, organ indices, senescence markers, antioxidant activity, neuronal integrity, and behavioral performance were assessed to evaluate the protective role of licorice water (LW) extract. Further, gut microbiota profiling, network pharmacology, and western blotting were employed to investigate further the potential mechanisms underlying the antiaging effects of LW.

**Results:**

LW administration significantly improved body weight gain, organ indices, and hippocampal structure in aging mice, increased antioxidant enzyme activity, and decreased the proportion of SA-β-Gal-positive cells. Moreover, LW treatment reshaped gut microbiota composition by lowering the *Firmicutes/Bacteroidota* (F/B) ratio and increasing the relative abundance of beneficial bacterial taxa. Network pharmacology analysis identified 66 licorice-associated antiaging genes, with quercetin, kaempferol, naringenin, formononetin, and licochalcone A as key active components. The principal molecular targets included AKT1, TP53, ESR1, CASPASE3, and BCL2, while the major enriched pathways involved PI3K–Akt, lipid and atherosclerosis, AGE–RAGE, MAPK, and IL-17 pathway. Furthermore, Western blot analysis revealed that LW significantly downregulated the expression of p-PI3K, p-AKT, and p-mTOR in brain tissue.

**Conclusion:**

These findings demonstrate that LW exerts protective antiaging effects in D-Gal–induced mice by enhancing antioxidant activities, safeguarding neuronal function to improve cognition, restoring gut microbiota balance, and modulating the PI3K/AKT/mTOR pathway, supporting its promise as a candidate for antiaging interventions.

## 1. Introduction

Aging is a complex biological process (BP) characterized by the progressive decline in tissues and organs' physiological function and structural integrity. It is a major risk factor for the onset of various related diseases, including Alzheimer's disease (AD) and Parkinson's disease (PD), both of which have shown rising incidence in the aging global population and impose significant socioeconomic burdens [1, 2]. Therefore, it is critical to elucidate the molecular mechanisms underlying aging and to develop safe and effective antiaging interventions.

Several herbal medicines, including ginseng, *Astragali radix*, *Lycium barbarum*, and licorice (*Glycyrrhiza uralensis*), have shown promising antiaging potential [3, 4]. Licorice root has a long history of use in traditional medicine systems across cultures. Licorice offers advantages for antiaging research compared with other traditional Chinese medicines, such as ginseng and *Astragali radix*. It is cultivated widely across Asia, Europe, North America, and other regions, providing an abundant supply of raw materials and ensuring a stable, sustainable source of supply [5]. Its cultivation cycle is considerably shorter than that of ginseng and similar herbs. At the same time, its cost is more economical, approximately half the price of Astragali radix and only one-fifth to one-tenth the cost of ginseng [6]. Its bioactive constituents—such as flavonoids, saponins, and polysaccharides—have been reported to exhibit antioxidant, immunomodulatory, detoxifying, and antiaging properties [7, 8]. While ancient texts like the *Divine Farmer's* Classic of Materia Medica (circa 200 ce) reference the longevity-promoting effects of licorice [5], such historical claims must be interpreted cautiously and require modern scientific validation.

Recent pharmacological studies have begun to substantiate licorice's antiaging effects. For instance, aqueous extracts of licorice were shown to attenuate D-galactose (D-Gal)-induced brain aging in rats by mitigating oxidative stress [9, 10]. Other investigations suggest that licorice modulates the taurine metabolic axis via upregulation of CDO1 and CSAD, implicating a systemic mechanism [11]. Additionally, ethanolic extracts of licorice increased the survival of *C. elegans* to exposed to oxidative stress, further supporting its antioxidant activity [12].

Accumulating evidence indicates that structural and functional alterations of the gut microbiota are closely linked to the aging process [13]. Aging profoundly reshapes the microbial composition: on one hand, the abundance of beneficial bacteria with anti-inflammatory and metabolic regulatory roles declines sharply. For instance, levels of probiotics such as *Bifidobacterium* and *Lactobacillus* are reported to be 30%–50% lower in elderly individuals compared with younger counterparts [14]. The proportion of harmful bacteria and opportunistic pathogens increases, with a significant rise in pro-inflammatory taxa, such as *Proteobacteria* and *Fusobacteria* [15]. This dysbiosis not only compromises intestinal barrier integrity and increases gut permeability but also facilitates the translocation of bacterial endotoxins into the bloodstream. These endotoxins, in turn, trigger systemic low-grade chronic inflammation, which further aggravates the decline in hepatic detoxification and hippocampal neuronal damage, thereby accelerating cognitive deterioration and multiorgan functional degeneration [16]. Current research on the modulatory effects of licorice on gut microbiota has primarily focused on immune enhancement and type 2 diabetes [17]. Therefore, the present study was designed to investigate whether licorice could regulate gut microbiota in aging mice.

Despite these findings, the precise molecular targets and signaling pathways through which licorice exerts its antiaging effects remain incompletely understood. Given the complexity of herbal medicines—with their multicomponent and multitarget nature—single-target approaches are insufficient. Network pharmacology has emerged as a powerful strategy to explore such complexity by integrating pharmacological data with systems biology. This approach facilitates the identification of compound–target–disease relationships, predicts mechanistic pathways, and improves drug development efficiency [18, 19]. It is increasingly applied in aging research and studies of age-related neurodegenerative diseases such as AD and PD [20, 21]. However, network pharmacology can only establish associative relationships between molecules and cannot directly confirm causal relationships; its prediction results rely on the integrity and accuracy of databases and are susceptible to the influence of false-positive data [22]. Therefore, experimental verification is required to ensure the scientific validity and reliability of research conclusions.

In this study, the antiaging effect of licorice water (LW) in D-Gal-induced aging mice was investigated, along with the changes in body weight, multiorgan senescence status, antioxidant capacity, hippocampal neuronal damage, and cognitive function after LW intervention. On the other hand, the alteration of gut microbiota in aging mice following LW administration was evaluated. The potential antiaging mechanisms of LW were predicted based on network pharmacology, and then verified by western blot. This work aims to support the future development of licorice-based antiaging therapeutics.

## 2. Materials and Methods

### 2.1. Reagents and Materials

Licorice was procured from Shennong Bencaotang (Baicheng) Pharmaceutical Co., Ltd. (Lot No. 20230011, Jilin Province, China) and authenticated by Professor Yugang Gao of Jilin Agricultural University (Changchun, Jilin, China). To prepare the LW extract, the raw material underwent three successive reflux extractions in distilled water at 100°C for 2 h each, followed by concentration using rotary evaporation and subsequent freeze-drying. Rabbit polyclonal antibodies p-AKT (AF0016), p-PI3K (AF3241), PI3K (AF6241), mTOR (AF6308), p-mTOR (AF3308), total AKT (AF6261), Nrf2 (AF0639), Keap1 (AF5266), and HO-1 (AF5393 were obtained from Affinity Biosciences Co., Ltd. (Liyang, Jiangsu, China). Other biochemical reagents, including catalase (CAT), superoxide dismutase (SOD), glutathione peroxidase (GSH-Px), malondialdehyde (MDA), total antioxidant capacity (T-AOC), and acetylcholinesterase (AChE) were sourced from Shanghai Enzyme-Linked Biotechnology Co., Ltd. (Shanghai, China). All other chemicals and reagents were purchased from Sigma–Aldrich (St Louis, MO, USA) and were of analytical grade.

### 2.2. Animals

ICR mice (20 ± 2 g) were obtained from Liaoning Changsheng Biotechnology Co., Ltd. (Liaoning, China) with an accompanying quality certificate (SCXK [Liao]-0001). The animals were maintained in a specific-pathogen-free facility under controlled environmental conditions: a 12-h light/dark cycle, temperature of 23 ± 2 °C, and relative humidity of 55% ± 5%. The Ethics Committee of Changchun University of Chinese Medicine reviewed and approved all experimental procedures involving animals following the Guidelines for the Care and Use of Laboratory Animals (Approval No. 2023311).

### 2.3. Animal Model and Drug Administration

A total of 40 ICR mice were randomly assigned to four groups, with 10 animals per group: a high-dose LW extract group (LW2), a low-dose licorice extract group (LW1), a control group (normal), and a D-Gal-induced aging group (D-Gal). After a 1-week acclimatization period, mice in the D-Gal, LW1, and LW2 groups received daily intraperitoneal injections of D-Gal at a dose of 700 mg/kg for 9 consecutive weeks, following a previously established protocol [23]. This dosage was selected based on previous studies demonstrating its effectiveness in inducing aging-like phenotypes in rodents while maintaining an acceptable safety profile [24]. Licorice extract was administered starting in the 5th week to simulate therapeutic intervention rather than preventive treatment. The LW doses (1 and 2 g/kg/day) were selected based on preliminary tolerability studies and published data on safe extract ranges without observed adverse effects [25]. Normal group mice received an equivalent volume of saline throughout the experimental period. Starting the fifth week, mice in the LW1 and LW2 groups were administered LW extract via intragastric gavage. The treatment regimens were as follows: Normal group, healthy mice receiving saline; D-Gal group, D-Gal-induced aging mice receiving saline; LW1 group, D-Gal-induced aging mice treated with 1 g/kg/day of LW; LW2 group, D-Gal-induced aging mice treated with 2 g/kg/day of LW. Body weights were recorded weekly. At the end of the 10-week experimental period, all animals were euthanized for further analysis. Brain tissues from each group were collected and fixed in 4% paraformaldehyde for histological examination. Furthermore, serum and organ samples were harvested and stored at −80°C for subsequent analyses.

### 2.4. Biochemical Analysis

To verify whether LW can enhance the antioxidant activity in aging mice, circulating levels of T-AOC, CAT, MDA, GSH-Px, and SOD were measured using ELISA kits (Enzyme-linked Biotechnology Co., Ltd., Shanghai, China).

### 2.5. H&E Staining

Brain tissue samples were dehydrated through a graded series of increasing ethanol concentrations, followed by xylene-based clearing, and then embedded in paraffin. The tissues were cut into 5-μm thick sections. The sections underwent a 20-min immersion in dewaxing solution I and another 20-min immersion in dewaxing solution II. They were then subjected to two 5-min washes in anhydrous ethanol I and II, followed by a 5-min soak in 75% ethanol, and rinsed in tap water. For staining, the sections were incubated for 5-min in hematoxylin dye, followed by a wash in tap water. The sections were then dehydrated through 5-min immersions in 95% and 85% ethanol, followed by a 5-min incubation in eosin dye. Afterward, dehydration was completed using xylene, and the sections were mounted with neutral resin and absolute ethanol. Finally, histopathological analysis was performed using an optical microscope (Leica RM2235, Wetzlar, Germany).

### 2.6. Nissl Staining

The sections were dewaxed with xylene and processed through a series of graded alcohol solutions before being cut into 6-μm thick slices. After a 20-min immersion in dewaxing solutions I and II, the sections were incubated for 5 min in 75% ethanol, followed by a rinse in tap water. The sections were then incubated for 3 min in Nissl staining solution and rinsed with distilled water. Dehydration was performed using 95% and the sections were ethanol, then mounted with neutral gum. Finally, the sections were examined under a Leica RM2235 microscope (Wetzlar, Germany).

### 2.7. SA-β-Gal Staining

Frozen tissue sections were first rewarmed at room temperature for 10 min. A sufficient volume of β-galactosidase staining fixative was then added dropwise to ensure complete coverage of the tissue, followed by fixation at room temperature for 20 min. After fixation, the sections were washed in PBS by immersion three times, each for 5 min. The sections were subsequently transferred to a light-proof humid chamber, where an appropriate amount of β-galactosidase staining working solution was added dropwise to fully cover the tissue. The humid chamber was incubated at 37°C, and color development was monitored under a microscope at 2-h intervals. Once staining was evident, the working solution was removed, and the sections were washed sequentially in PBS twice and then in distilled water twice. The sections were dehydrated in absolute ethanol twice (3 min each), cleared in xylene three times (2 min each), and finally mounted with neutral balsam. The prepared sections were then examined under a microscope to evaluate the staining results.

### 2.8. Quantitative Real-Time PCR (qRT-PCR)

Total RNA was extracted using the Trizol reagent (CoWin Biosciences, China), and the RNA concentration was determined using a spectrophotometer (Thermo Fisher Scientific, China). Complementary DNA (cDNA) was then synthesized by reverse transcription with the PrimeScript RT reagent Kit (TransGen Biotech, China). qRT-PCR was subsequently performed using the SYBR Green PCR Kit (TransGen Biotech, China). Amplification and detection were conducted on a LightCycler 96 System (Roche, Germany) to quantify the expression of *P16* and *P21*, with *GAPDH* serving as the internal reference gene. The primer sequences were as follows: *P16* forward 5′-CGCAGGTTCTTGGTCACTG-3′, reverse 5′-TGTTCACGAAAGCCAGAGCG-3′; *P21* forward 5′-GTACTTCCTCTGCCCTGCTG-3′, reverse 5′-AATCTGTCAGGCTGGTCTGC-3′. Each sample was analyzed in triplicate, and the relative mRNA expression levels of *P16* and *P21* were calculated using the 2^−ΔΔCt^ method.

## 3. Behavioral Experiments

The Morris water maze (MWM) was used to evaluate spatial learning and memory in mice over 6 days, consisting of place navigation and spatial probe tests. The place navigation task was conducted during the first 5 days. Each mouse was released into the pool facing the wall, and its swimming trajectory was automatically recorded and analyzed using the water maze camera system. The swimming distance and the time taken to locate and remain on the hidden platform were defined as the escape latency. Once a mouse climbed onto the submerged platform and remained for 10 s, video tracking and data collection were automatically stopped. If the mouse failed to find the platform within 120 s, it was gently guided to it with a wooden stick and allowed to remain there for 10 s; in such cases, the escape latency was recorded as 120 s. On the 6th day, the spatial probe test was performed. The platform was removed, and each mouse was released from the quadrant opposite to the previous platform location. The swimming trajectory within 120 s, the number of times the mouse crossed the original platform area, and the swimming duration in the target quadrant were recorded. All behavioral data were analyzed using Tracking Master animal behavior analysis software.

### 3.1. DNA Isolation and Gut Microbiota Sequencing

Fecal samples from the normal, D-Gal, and LW2 mice were flash frozen in liquid nitrogen and stored at −80°C. Genomic DNA was isolated from the feces using the CTAB/SDS protocol, and its quality was assessed by electrophoresis on a 1% agarose gel. The V3–V4 region of the 16S ribosomal DNA (rDNA) was amplified using primers 341F (CCTACGGGNGGCWGCAG) and 806 R (GGACTACHVGGGTATCTAAT). 16S rDNA sequencing was performed on an Illumina PE250 platform by Bioprofile Biotechnology Co., Ltd. (Shanghai, China). Operational taxonomic units (OTUs) were defined at a 97% similarity threshold. Data analysis and visualization were conducted using RStudio (RStudio, Boston, MA, USA) and Simca 14.1 (Umetrics, Sweden).

### 3.2. Network Pharmacology Analysis

Active compounds and corresponding targets related to licorice were identified using the Traditional Chinese Medicine Systems Pharmacology Database and Analysis Platform (TCMSP, http://tcmspw.com/tcmsp.php) by searching the keyword “licorice” and applying the filters of oral bioavailability (OB) ≥ 30% and drug-likeness (DL) ≥ 0.18 [26]. Candidate aging-related gene targets were obtained by searching the DisGeNET (https://www.disgenet.org/home/), Online Mendelian Inheritance in Man (OMIM, https://omim.org/), and GeneCards (https://www.genecards.org/) databases using the keyword “anti-aging” [27]. To identify shared targets between licorice and antiaging, a Venn diagram was generated using the Draw Venn Diagram tool (http://bioinformatics.psb.ugent.be/webtools/Venn/) [28]. The overlapping targets between licorice-derived active ingredients and aging-related genes were imported into Cytoscape (version 3.10.1) to construct a network correlation [29]. The shared targets were submitted to the STRING database (https://cn.string-db.org/) for protein–protein interaction (PPI) analysis, specifying *Homo sapiens* as the species and applying the highest confidence score threshold (>0.9) [30]. Gene Ontology (GO) and Kyoto Encyclopedia of Genes and Genomes (KEGG) pathway enrichment analyses were conducted using the DAVID database (https://david.ncifcrf.gov/) to explore the biological functions of the identified gene targets. The KEGG pathway enrichment was filtered using a *p*-value threshold of <0.05, indicating a commonly accepted statistical significance for biological pathways [31]. Targets were filtered using DAVID's default functional annotation chart algorithm. The top 20 significantly enriched networks were visualized based on *p*-value ranking from the enrichment analysis [32].

### 3.3. Western Blotting

Western blot analysis was performed according to a previously described protocol [33]. Briefly, brain tissue was lysed using RIPA lysis buffer, and protein extraction was followed by quantification using a bicinchoninic acid assay kit (Pierce, Rockford, IL, USA). Approximately, 15 μg of protein was separated by electrophoresis on a 10% sodium dodecyl sulfate-polyacrylamide gel, then transferred to a polyvinylidene fluoride membrane. The membrane was incubated overnight at 4°C with primary antibodies against total PI3K, p-mTOR, HO-1, Keap1, Nrf2, AKT, mTOR, and p-AKT (all at 1:1000 dilution), followed by a 1-h incubation with a secondary sheep anti-rabbit antibody (1:3000) conjugated to horseradish peroxidase. Protein bands were visualized using enhanced chemiluminescence and quantified using ImageJ software.

### 3.4. Statistical Analysis

All data analyses were performed using SPSS (version 16.0, IBM, Armonk, NY, USA), and the results are presented as the mean ± standard deviation (SD). Intergroup comparisons were conducted using analysis of variance (ANOVA), followed by Tukey's post hoc test to correct for multiple comparisons. Pearson's correlation analysis was carried out using the Chiplot program (https://www.chiplot.online/). A *p*-value of less than 0.05 was considered statistically significant at a 95% confidence level.

## 4. Results

### 4.1. Effect of LW on Body Weight and Senescence of Liver, Kidney, Heart, and Spleen in Aging Mice

**Figure 1:**
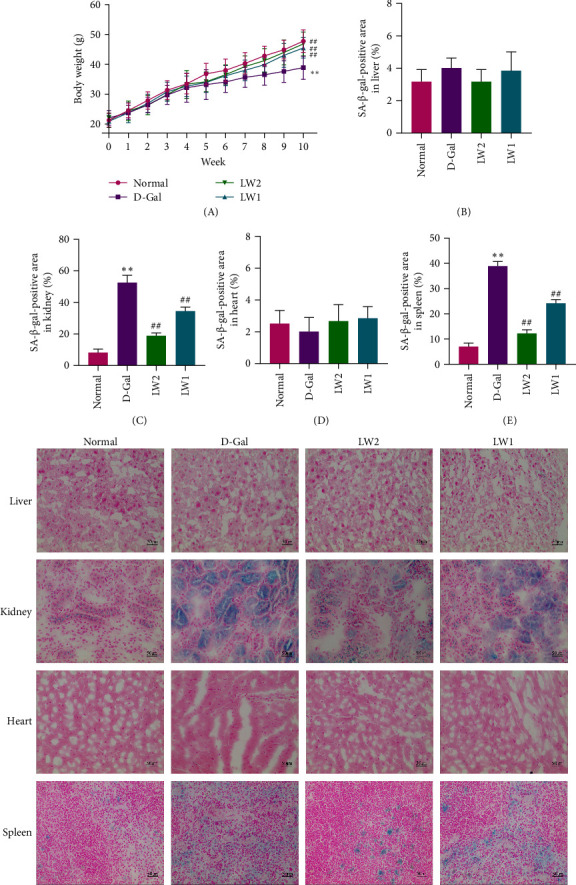
Impacts of LW on body weight and senescence of liver, kidney, heart, and spleen in aging mice. (A) Body weight. (B–E) Effects of LW on the percentages of SA-β-Gal-positive cells in liver, kidney, heart, and spleen. *⁣*^*∗*^*p* < 0.05 and *⁣*^*∗∗*^*p* < 0.01 compared with the normal group, ^#^*p* < 0.05 and ^##^*p* < 0.01 compared with the D-Gal group.

Body weight serves as a reliable indicator of general physiological development. Throughout the experimental period, mice were weighed weekly (Figure 1A). During the initial 4 weeks, there were no statistically significant differences in mean body weight among the four groups (*p* > 0.05). However, as the study progressed, mice treated with D-Gal showed a slower rate of weight gain. About 6 weeks of LW extract intervention markedly mitigated the adverse effects of D-Gal. By the end of the 10-week treatment period, the LW2 group demonstrated weight gain comparable to that of the control group and significantly greater than that of the D-Gal group (*p*  < 0.01). Furthermore, organ indices were calculated for the brain, liver, spleen, heart, and kidneys (Table 1), reflecting these organs' functional status and general health. LW treatment significantly improved organ indices across all examined tissues compared to the D-Gal group.

Cellular senescence was assessed by SA-β-Gal staining (Figure 1). In comparison to the normal control group, the proportion of SA-β-Gal-positive cells in the kidneys and spleens was significantly elevated in the D-gal group, confirming that D-Gal effectively induced multiorgan aging in the experimental animals. Following treatment with LW extract, the percentages of SA-β-Gal-positive cells in both the LW2 and LW1 groups were significantly reduced, indicating that LW intervention mitigated D-Gal–induced senescence in multiple organs of mice.

### 4.2. LW Increased the Antioxidant Capacity in Aging Mice

**Figure 2:**
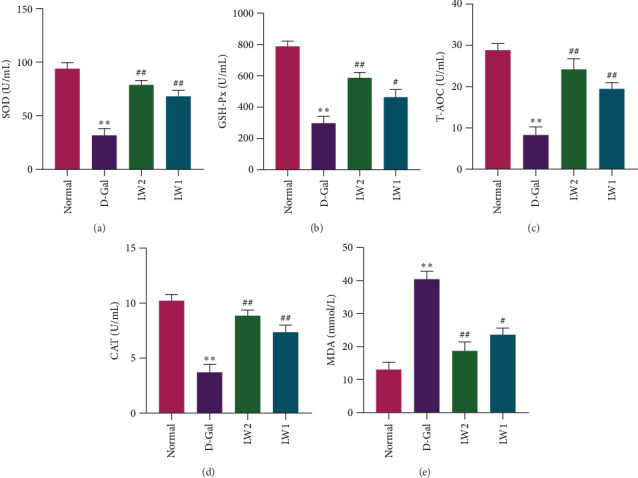
Effects of LW on antioxidant capacity in aging mice. (A–E) Effects of LW on serum levels of SOD, CAT, GSH-Px, T-AOC, and MDA (*n* = 10). *⁣*^*∗*^*p* < 0.05 and *⁣*^*∗∗*^*p* < 0.01 compared with the normal group, ^#^*p* < 0.05 and ^##^*p* < 0.01 compared with the D-Gal group.

**Figure 3:**
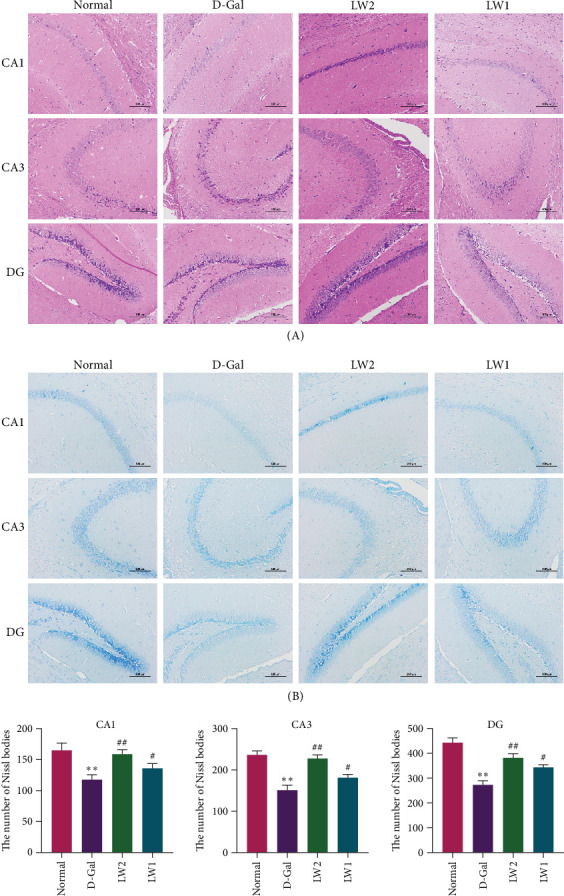
LW abrogated brain damage in aging mice. (A) Hematoxylin and eosin staining of the cornu ammonis 1 (CA1), cornu ammonis 3 (CA3), and dentate gyrus (DG). (B) Nissl staining of the cornu ammonis 1 (CA1), cornu ammonis 3 (CA3), and dentate gyrus (DG). *⁣*^*∗*^*p* < 0.05 and *⁣*^*∗∗*^*p* < 0.01 relative to normal mice, ^#^*p* < 0.05 and ^##^*p* < 0.01 relative to the D-Gal mice.

Mitochondrial oxidative phosphorylation is a primary source of reactive oxygen species (ROS). Excessive ROS generation leads to oxidative stress in the brain, causing mitochondrial damage and functional impairments [34]. Mitochondrial dysfunction is a key driver of the aging process [35]. In this study, D-Gal administration significantly reduced the activities of T-AOC, GSH-Px, CAT, and SOD compared to the control group (*p* < 0.05) (Figure 2A–E). At the same time, it elevated MDA levels (Figure 3F) (*p* < 0.05). Treatment with LW extract for 6 weeks significantly increased antioxidant enzyme activities (T-AOC, GSH-Px, CAT, and SOD) (*p* < 0.05) and concurrently reduced MDA levels, particularly in the LW2 group. These findings suggest that LW effectively counteracts D-Gal-induced oxidative stress in aging mice.

### 4.3. LW Improved Neural Damage and Memory Deficit in Aging Mice

Hippocampal oxidative damage is pivotal in D-Gal-induced aging, contributing to neuronal loss and structural deterioration [36]. H&E staining (Figure 3A) revealed that hippocampal neurons in normal mice were densely packed and displayed typical morphology. In comparison, the D-Gal group showed marked neuronal loss, disrupted organization, and scattered distribution, accompanied by an increased presence of degenerated and necrotic cells. To further assess neuronal integrity, Nissl staining was performed (Figure 3B), highlighting a significant reduction in Nissl body density in D-Gal-treated mice. The LW administration substantially mitigated these effects. In the LW2 group, neurons revealed a considerable increase in Nissl bodies, which were more regularly arranged, indicating a restorative effect of LW on hippocampal morphology and neuronal viability.

**Figure 4:**
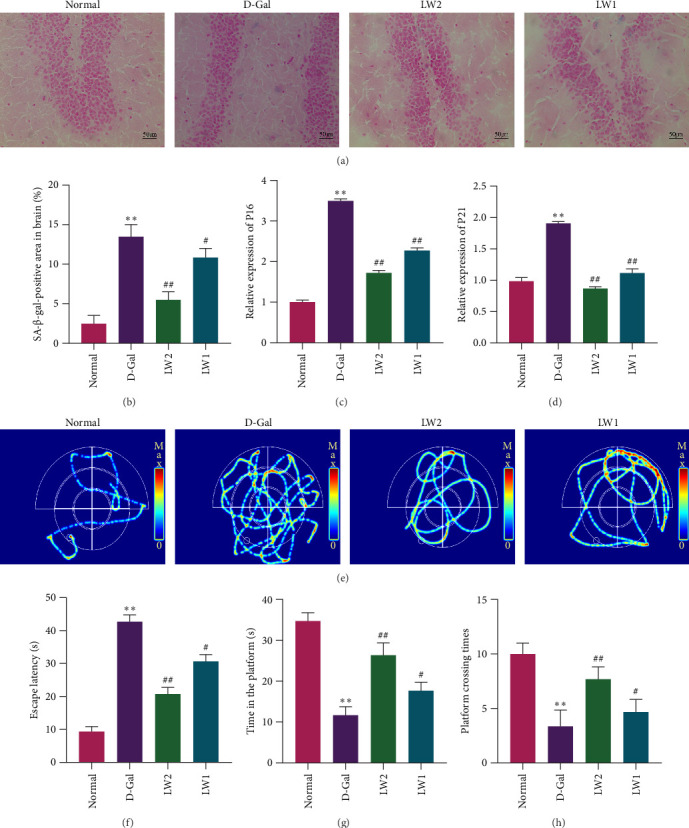
LW delayed brain aging and improved memory deficit in aging mice. (A, B) Representative images and the proportion of SA-β-gal-positive cells in brain tissue. (C, D) Relative expression of *P16* and *P21* in brain tissue. (E) Heat maps of spatial navigation in the MWM test. (F) The escape latency in the spatial exploration of the MWM test. (G) Time in the platform. (H) Platform crossing times. *⁣*^*∗*^*p* < 0.05 and *⁣*^*∗∗*^*p* < 0.01 relative to normal mice, ^#^*p* < 0.05 and ^##^*p* < 0.01 relative to the D-Gal mice.

The senescent state of brain tissue was assessed by SA-β-gal staining (Figure 4A,B) and qRT-PCR analysis (Figure 4C,D). SA-β-gal staining revealed that, compared with the model group, the proportion of SA-β-gal-positive cells in brain tissue was significantly reduced following LW administration, displaying a clear dose-dependent trend. Similarly, qRT-PCR results showed that mRNA expression levels of *P21* and *P16* were significantly elevated in the D-gal group, whereas LW treatment effectively decreased the expression of both genes. These results suggest that LW can attenuate brain tissue senescence in aging mice.

Cognitive function was further evaluated using the MWM (Figure 4E–H). In the place navigation task, mice in the D-gal group showed significantly prolonged escape latency compared with the normal control group. LW intervention significantly shortened the escape latency in aging mice. During the spatial probe trial, the D-gal group revealed a significant decline in the number of platform crossings and the time spent in the target quadrant compared to the controls. In comparison, LW treatment substantially increased both parameters. These results suggest that the LW intervention effectively improves spatial learning and memory capacity in aging mice.

### 4.4. LW-Mediated Actions on the Gut Microbiota in Aging Mice

**Figure 5:**
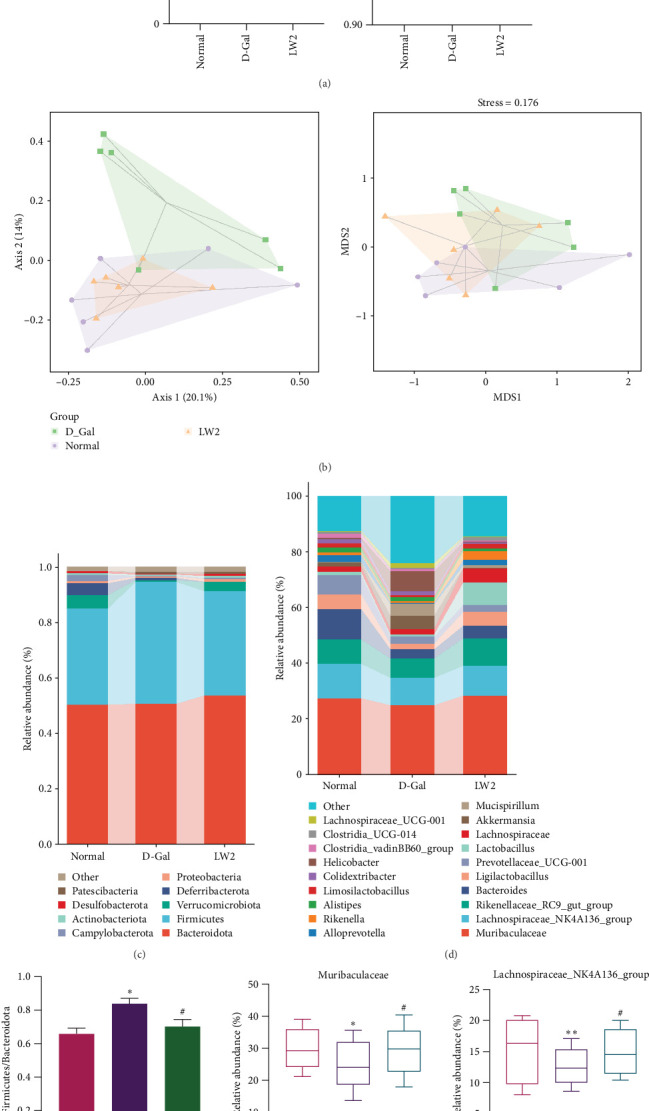
LW augmented gut microbiota composition and diversity in aging mice. (A) α-Diversity, as evidenced by the Good's coverage, Chao 1, observed_species, Shannon, and Simpson indices. (B) Score plots from principal coordinate (PCoA) and nonmetric multidimensional scaling (NMDS) analyses. (C) Gut microbiota composition at the phylum and (D) genus levels. The *Firmicutes*/*Bacteroidota* ratio (E). Abundances of *Muribaculaceae* (F), *Lachnospiraceae*_NK4A136_group (G), *Rikenellaceae*_RC9_gut_group (H), *Bacteroides* (I), and *Ligilactobacillus* (J). *⁣*^*∗*^*p* < 0.05 and *⁣*^*∗∗*^*p* < 0.01 relative to normal mice, ^#^*p* < 0.05 and ^##^*p* < 0.01 relative to the D-Gal mice.

Fecal samples were collected from normal, D-Gal, and LW2 mice for the analysis of 16s rDNA. The Good's coverage index exceeded 0.98 across all groups (Figure 5A), indicating that the sequencing depth was sufficient to capture the majority of microbial diversity, with no significant differences between groups (*p* > 0.05). Similarly, no significant differences in α-diversity (Chao 1, observed_species, Shannon, and Simpson indices) were observed among the groups, suggesting comparable within-sample diversity. In comparison, β-diversity analyses revealed clear group compositional differences (Figure 5B). Principal coordinate analysis (PCoA) and nonmetric multidimensional scaling (NMDS) plots demonstrated a distinct separation between the D-Gal and normal groups, indicating a marked shift in microbial community structure following D-Gal administration. This was supported by PERMANOVA analysis (*R*^2^ = 0.321, *p*=0.001), confirming that the differences in community composition were statistically significant. Interestingly, the LW2 group clustered more closely with the normal group, suggesting that LW2 administration partially restored the gut microbiota composition in aged mice.

Furthermore, we assessed the relative abundance of major bacterial phyla. *Bacteroidota*, *Firmicutes*, and *Verrucomicrobiota* dominated all groups (Figure 5C). In normal mice, their respective abundances were 50.32%, 34.75%, and 4.73%. D-Gal treatment reduced *Verrucomicrobiota* to 0.06% and increased *Firmicutes* to 43.89%. LW2 administration reversed these trends, increasing *Bacteroidota* and *Verrucomicrobiota* to 53.63% and 0.32% while reducing Firmicutes to 37.59%. Notably, the *Firmicutes/Bacteroidota* (F/B) ratio, which is known to be altered with aging, increased in the D-Gal group (0.87) compared to normal (0.69) and was reduced to 0.70 with LW2 treatment (Figure 5E).

At the genus level (Figure 5D), the most abundant taxa included *Muribaculaceae*, *Lachnospiraceae_NK4A136_group*, *Rikenellaceae_RC9_gut_group*, *Bacteroides*, and *Ligilactobacillus*. D-Gal administration markedly reduced the relative abundance of these genera (Figure 5). At the same time, LW2 treatment significantly restored their levels, highlighting the modulatory effect of LW2 on gut microbiota composition in aged mice.

### 4.5. Analysis of the Potential Antiaging Signaling Pathways of LW by Network Pharmacology

**Figure 6:**
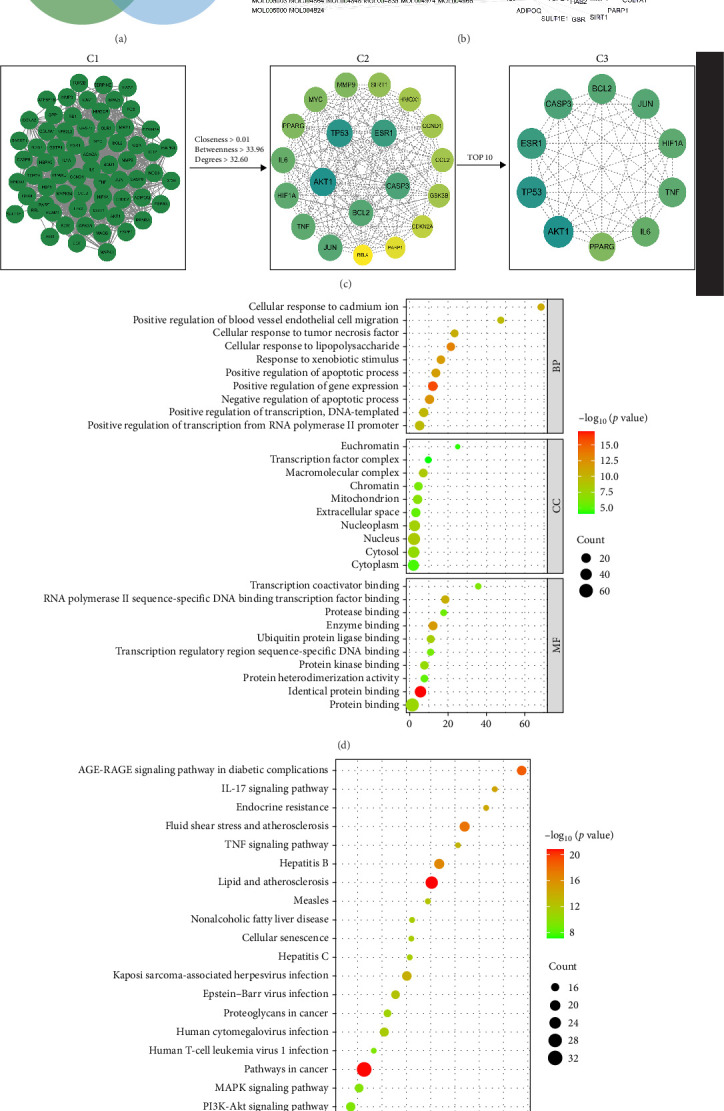
Network pharmacology analysis of the antiaging effects of LW. (A) Venn diagram depicting the overlap between predicted targets of licorice (230 total) and antiaging-related targets (638 total), identifying 66 intersecting targets. (B) Compound-target network. The network includes 158 nodes (92 compound-nodes represented as green circles and 66 target nodes represented as purple squares) and 517 edges. (C) PPI (protein–protein interaction) network of the 66 overlapping targets. (C1) displays the overall PPI network; (C2) highlights key nodes filtered by topological parameters (closeness > 0.01, betweenness > 23.96, and degree > 32.60); and (C3) shows the top 10 hub genes based on degree centrality. Node size reflects the degree, and edge thickness corresponds to the confidence in interaction. (D) Gene Ontology (GO) enrichment analysis of the 66 intersecting targets, covering three categories: biological processes (BPs), cellular components (CCs), and molecular functions (MFs). The top 10 significantly enriched terms in each category are displayed. Bubble size indicates the number of genes involved in each term, and color represents the statistical significance (−log_10_ (*p*-value)). (E) KEGG pathway enrichment analysis. A total of 142 pathways were enriched (*p* < 0.05), with the top 20 shown. The *x*-axis indicates the enrichment ratio, bubble size corresponds to the number of associated genes, and color denotes −log_10_ (*p*-value).

The TCMSP database was screened, and 92 active ingredients and 230 corresponding target genes were identified from licorice. By screening the DisGeNET, GeneCards, and OMIM databases and eliminating duplicate targets, 638 targets associated with antiaging were identified. Finally, 66 overlapping protein targets were identified that are shared between licorice and antiaging mechanisms through intersection analysis (Figure 6A). The licorice active ingredients and their intersecting targets were imported into Cytoscape 3.9.10 to construct a compound–target interaction network (Figure 6B) The top 10 key compounds identified were quercetin, kaempferol, naringenin, formononetin, licochalcone A, isorhamnetin, Glepidotin A, 1-Methoxyphaseollidin, 7-Methoxy-2-methyl isoflavone and Gancaonin G. The PPI network was constructed using STRING version 11.0 resulting in a network comprising 66 nodes and 1076 edges (Figure 6C). Topological analysis was conducted using the Network Analyzer tool in Cytoscape, and 20 core targets were identified based on threshold values for betweenness (≥33.96), Closeness (≥0.010), and degree (≥32.60). Among these, the top 10 hub targets were AKT1, TP53, ESR1, CASPASE3, BCL2, JUN, TNF, HIF1A, IL-6, and PPARG.

GO and KEGG enrichment analyses were performed on the 66 identified targets. GO analysis revealed a total of 422 BP terms, 43 cellular component (CC) terms, and 85 molecular function (MF) terms, all with statistical significance (*p* < 0.05). The top 10 enriched GO terms are shown in Figure 6D. Key BP enrichments included positive regulation of gene expression, cellular response to lipopolysaccharide, and negative regulation of apoptosis. In the CC category, targets were mainly associated with the macromolecular complex, nucleus, and nucleoplasm. MF terms were predominantly enriched in protein binding, enzyme binding, RNA polymerase II-specific DNA binding, and transcription factor binding. KEGG pathway analysis identified 142 significantly enriched pathways (*p* < 0.05), as illustrated in Figure 6E. These findings suggest that licorice exerts its antiaging effects primarily through the PI3K-Akt pathway, as well as pathways related to lipid metabolism, atherosclerosis, cancer, the AGE-RAGE pathway in diabetic complications, MAPK, and the IL-17 pathway.

### 4.6. LW Promotes the PI3K/AKT/mTOR Axis in Aging Mice

**Figure 7:**
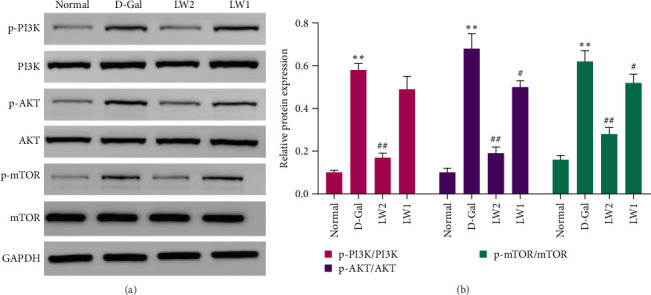
The effects of LW treatment on the PI3K/AKT/mTOR axes signaling pathway in D-Gal-induced aging mice. (A) Representative western blot images of PI3K, p-PI3K, AKT, p-AKT, mTOR, and p-mTOR protein expression levels. GAPDH was used as a loading control. (B) Quantifying phosphorylated-to-total protein ratios (p-PI3K/PI3K, p-AKT/AKT, and p-mTOR/mTOR). Data are presented as mean ± SD.*⁣*^*∗*^*p* < 0.05 and *⁣*^*∗∗*^*p* < 0.01 vs. Normal group; ^#^*p* < 0.05 and ^##^*p* < 0.01 vs. D-Gal group.

The network pharmacology analysis suggested that the PI3K/AKT signaling pathway may underlie the antiaging effects of licorice. To confirm this hypothesis, Western blot analysis was performed on brain tissue samples (Figure 7A,B). D-Gal treatment markedly increased the phosphorylated mTOR, AKT, and PI3K protein expression levels (*p* < 0.05), indicating pathway activation. LW administration significantly suppressed these phosphorylated proteins in a dose-dependent manner, supporting the regulatory role of LW on the PI3K/AKT pathway during aging.

## 5. Discussion

Population aging is a pressing global issue, with projections indicating that the population aged ≥60 years will each 2.1 billion by 2050 [37]. Although aging is a natural and inevitable BP characterized by a progressive decline in physiological functions and increased disease susceptibility, emerging evidence suggests that certain interventions may attenuate its deleterious effects. This study systematically verified the antiaging impact of LW by investigating physiological status, oxidative stress, neurofunction, gut microbiota, and potential antiaging mechanisms.

Body weight served as a macroscopic indicator of systemic metabolism and overall health status. Mice in the D-Gal group exhibited significantly slower body weight gain during the later stages, a change associated with impaired energy utilization resulting from glucose metabolic disturbance and progressive organ dysfunction [38]. In comparison, LW treatment, particularly in the LW2 group, reversed this decline, restoring body weight gain to levels comparable to those of the normal control group. Moreover, LW significantly increased the organ indices of the liver, kidney, heart, and spleen, parameters that directly reflect organ development and functional integrity. These findings suggest that LW supports the maintenance of normal organ physiology by improving metabolic efficiency and mitigating organ injury.

The SA-β-gal staining, a classical marker of cellular senescence, further confirmed these protective effects [39]. The proportion of SA-β-gal-positive cells in the kidney and spleen was significantly elevated in the D-Gal group, validating the successful induction of multiorgan senescence. Following LW administration, the proportion of positive cells was significantly reduced, indicating that LW protects multiple organs against D-Gal–induced senescent injury by suppressing the cellular senescence process.

Excessive accumulation of ROS triggers a variety of diseases, including cancer [40] and aging [41], among others. ROS can induce mitochondrial damage and lipid peroxidation (as reflected by elevated MDA levels) while simultaneously suppressing the activity of key antioxidant enzymes such as SOD, CAT, and GSH-Px. This establishes a vicious cycle of “oxidative stress–mitochondrial dysfunction–accelerated aging.“ In the present study, the activities of T-AOC, SOD, CAT, and GSH-Px were significantly reduced in the D-Gal group, accompanied by a significant rise in MDA content. However, LW intervention effectively reversed these changes, suggesting that LW improves the antioxidant defense system, scavenges excess ROS, and mitigates lipid peroxidation injury.

The hippocampus, a critical brain region for learning and memory, is highly vulnerable to structural and functional impairment under aging conditions [42]. Neuronal degeneration, loss, and disorganization are significant contributors to cognitive decline in D-Gal–induced aging mice. Histological analysis in this study revealed that hippocampal neurons in the D-Gal group were sparse and exhibited increased necrosis, as observed on H&E staining. In comparison, the Nissl staining showed a reduced density of Nissl bodies, a morphological hallmark of neuronal function. In comparison, LW treatment restored neuronal density, increased the number of Nissl bodies, and preserved their orderly arrangement, providing direct evidence of its neuroprotective effect in aging mice.

The SA-β-gal staining demonstrated that LW reduced the proportion of senescent cells in brain tissue. Consistently, the qPCR analysis revealed that LW downregulated the mRNA expression of senescence-associated genes *P21* and *P16*. These genes function as key tumor suppressors in cell cycle regulation, and their overexpression induces cell cycle arrest and accelerates cellular senescence [43]. Behavioral assessment using the MWM further showed that LW significantly shortened escape latency, increased platform crossing frequency, and prolonged the duration spent in the target quadrant. These results suggest that LW improves the cognitive capacity of aging mice by mitigating neuronal damage and slowing neural senescence.

Gut microbiota dysbiosis is a hallmark of aging and is strongly linked to systemic oxidative stress, organ senescence, and neurofunctional decline [44]. Microbiota analysis revealed that LW effectively ameliorated D-Gal–induced dysbiosis, restoring the F/B ratio and enriching beneficial bacterial taxa, such as *Muribaculaceae*, *Lachnospiraceae* NK4A136 group, and *Ligilactobacillus*. These genera are associated with anti-inflammatory, antioxidant, and neuroprotective functions [45–47]. Hence, the microbiota–gut–brain axis may partially mediate the antiaging effects of LW.

To further explore underlying mechanisms, a combination of network pharmacology and experimental validation was employed. Using the TCMSP database, 92 active components of licorice and 230 corresponding targets were identified. Intersecting these with 638 known antiaging targets yielded 66 core targets. The construction of a “component–target” interaction network highlighted quercetin, kaempferol, and related compounds as the principal active ingredients. PPI network analysis further identified 10 hub targets, including AKT1 and TP53. KEGG enrichment analysis revealed that the PI3K–Akt pathway was among the key regulatory pathways.

Western blot analysis was then conducted to validate these network-based predictions. Results showed that expression levels of phosphorylated PI3K, AKT, and mTOR were significantly elevated in the brains of D-Gal–treated mice. Overactivation of the PI3K/AKT/mTOR pathway is known to promote cellular senescence, suppress autophagy, and aggravate neuronal injury. In comparison, LW significantly reduced the expression of these phosphorylated proteins, inhibiting excessive pathway activation. These findings confirm that LW mitigates D-Gal–induced senescence through negative regulation of the PI3K/AKT/mTOR axis, in agreement with the predictions derived from network pharmacology.

Although this study has achieved some research findings, it also has several limitations. First, network pharmacology is inherently predictive and may yield false positives or overlook context-specific interactions. Second, while mouse models provide valuable insights, physiological differences from humans, especially in aging and microbiota composition, limit translational applicability. Third, licorice is a complex herbal mixture, and the lack of standardization in compound concentrations may introduce variability in efficacy. Lastly, the observed pathway modulation reflects the pathological context of D-Gal-induced aging; effects in normal aging or other aging models require further exploration.

## 6. Conclusion

In this study, we investigated the antiaging effect of licorice and its potential mechanisms. The results showed that LW exerted a notable antiaging effect in D-Gal-induced aging mice. It alleviated slow body weight gain, improved the senescent state of multiple organs, including the liver, kidneys, heart, and spleen, and enhanced antioxidant capacity in aging mice. Meanwhile, LW protected hippocampal neurons, improved spatial learning and memory abilities, and restored the gut microbiota balance disrupted by D-Gal. Mechanistic studies revealed that LW could dose-dependently inhibit the PI3K/AKT/mTOR signaling pathway. In conclusion, LW exerts its antiaging effect by enhancing antioxidant capacity, protecting neurons, regulating gut microbiota, and modulating signaling pathways. And this research provides a reference for the development of licorice-related antiaging products.

## Figures and Tables

**Table 1:** Effects of LW on organ indices (% of body weight) in aging mice.

Group	Organ indices (%)			
	Liver	Kidney	Heart	Spleen
Normal	4.96 ± 0.20	1.63 ± 0.12	0.60 ± 0.06	0.30 ± 0.04
D-Gal	3.32 ± 0.15^*∗∗*^	1.32 ± 0.12^*∗∗*^	0.41 ± 0.03^*∗∗*^	0.24 ± 0.06^*∗*^
LW2	4.39 ± 0.19^#^	1.47 ± 0.11^#^	0.51 ± 0.03^#^	0.29 ± 0.07^#^
LW1	3.99 ± 0.29	1.39 ± 0.14	0.46 ± 0.04	0.27 ± 0.05

*Note*: Data were presented as mean ± standard deviation (*n* = 10). The organ index was calculated as follows: organ index (%)=(organ weight/body weight) × 100. *⁣*^*∗*^*p* < 0.05, *⁣*^*∗∗*^*p* < 0.01 vs. normal group, ^#^*p* < 0.05, ^##^*p* < 0.01 vs. D-Gal group.

## Data Availability

Data are contained within the article.
